# Special Issue: “Neurodegenerative Diseases: Recent Advances and Future Perspectives”

**DOI:** 10.3390/biomedicines12051080

**Published:** 2024-05-13

**Authors:** Inés López-Cuenca, Rosa De Hoz

**Affiliations:** 1 Ramon Castroviejo Institute for Ophthalmic Research, Complutense University of Madrid, 28040 Madrid, Spain; inelopez@ucm.es; 2 Health Research Institute of the Hospital Clínico San Carlos (IdISSC), 28040 Madrid, Spain

Neurodegenerative diseases include a heterogeneous group of conditions that pose a growing challenge to public health and the scientific community [1]. As the worldwide population ages, the impact of these diseases is increasing [2], underscoring the urgent need to improve our understanding of them. In all of the publications in this Special Issue, we have taken an in-depth look at the latest advances in the understanding, diagnosis and treatment of these complex diseases that affect millions of people worldwide [3].

In this Special Issue, we have compiled innovative research covering various aspects related to neurodegenerative diseases: advances in monitoring the progression of Huntington’s disease and multiple sclerosis [4], therapies aimed at improving executive function in mild cognitive impairment (MCI), as well as ocular imaging techniques for diagnosis and monitoring, and risk factor analysis [5]. Literature reviews have also been included to update the materials used in peripheral nervous system therapies, neuroprotective compounds, as well as the most advanced techniques in animal model research [6].

From these studies, it is essential to identify biomarkers and early diagnostic methods [7]. It is crucial to intervene before symptoms arise or in the early stages of these diseases, when therapeutic interventions can be most effective [8]. Research in this area is advancing rapidly, and the studies presented here offer promising prospects for the development of more accurate and non-invasive diagnostic tools.

In addition, tools that allow the monitoring of neurodegenerative pathology, such as the Total Functional Capacity score in Huntington’s disease, are indispensable. Another area of interest in the Special Issue is the exploration of risk factors associated with the development of these pathologies, such as depression, medication use and genetics.

We should not ignore the research on various therapeutic pathways compiled in this Special Issue, such as the use of piezoelectric materials or the neuroprotective potential of fisetin [9].

As the heterogeneity of the publications included here demonstrates, these diseases can be studied from a variety of angles, underscoring the importance of fostering interdisciplinary collaboration and knowledge sharing within the scientific community.

In summary, this Special Issue of *Biomedicines* provides a comprehensive and up-to-date overview of the latest advancements in the field of neurodegenerative diseases. Although we recognize that much work remains to be done, we trust that the knowledge and ideas presented here will serve as a platform for future research and interdisciplinary collaborations, with the aim of achieving a better understanding and treatment of these diseases.

We express our sincere gratitude to all the authors, reviewers, and contributors who have made this Special Issue possible. Their dedication and contributions are essential to advancing scientific knowledge and improving the quality of life of those affected by neurodegenerative diseases.

We hope that readers will find these papers inspiring and that they will spur others on to carry out further research and discovery in this crucial field.
